# A Strategic Imperative for Promoting Hospital Branding: Analysis of Outcome Indicators

**DOI:** 10.2196/14546

**Published:** 2020-01-22

**Authors:** Gow-Jen Shieh, Shi-Liang Wu, Che-Fu Tsai, Chi-Sen Chang, Tsung-Hung Chang, Ping-Wing Lui, Yuh Yao, Wayne Huey-Herng Sheu

**Affiliations:** 1 Department of Top Hospital Administration Taichung Veterans General Hospital Taichung Taiwan; 2 Department of Medicine, School of Medicine National Yang-Ming University Taipei Taiwan; 3 School of Medicine National Defense Medical Center Taipei Taiwan; 4 Institute of Medical Technology, College of Life Science National Chung-Hsing University Taichung Taiwan

**Keywords:** social media, branding, Facebook, Taiwan, health services research, marketing of health services

## Abstract

**Background:**

Optimizing the use of social media to promote hospital branding is important in the present digital era. In Taiwan, only 51.1% of hospitals have official Facebook fan pages. The numbers of likes for these hospitals are also relatively low.

**Objective:**

Our objective was to establish a special branding team for social media operation, led by top administrators of our hospital. Here we present our strategic imperative for promoting hospital branding as well as an analysis of its effectiveness.

**Methods:**

Led by top administrators, the branding team was formed by 11 divisions to create branding strategies. From 2016 to 2018, the team implemented action plans. All information unique to the hospital was posted on Facebook, as well as on the hospital’s official website. To determine the plans’ efficiencies, we obtained reference data from Google Analytics, and we compared Facebook Insights reports for 2016 with those for 2017 and 2018.

**Results:**

One of the branding team’s main missions was to establish branding strategies and to integrate segmental branding messages. In each quarter we regularly monitored a total of 52 action plan indicators, including those for process and outcome, and discussed the results at team meetings. We selected 4 main performance outcome indicators to reflect the effectiveness of the branding efforts. Compared with 2016, the numbers of likes posted on the Facebook fan page increased by 61.2% in 2017 and 116.2% in 2018. Similarly, visits to the hospital website increased by 4.8% in 2017 and 33.1% in 2018. Most Facebook fan page and website viewers were in 2 age groups: 25 to 34 years, and 35 to 44 years. Women constituted 60.42% (14,160/23,436) of Facebook fans and 59.39% (778,992/1,311,605) of website viewers. According to the Facebook Insights reports, the number of likes and post sharing both increased in 2017 and 2018, relative to 2016. Comment messages also increased from 2016 to 2018 (*P*=.02 for the trend). The most common theme of posts varied over time, from media reports in 2016, to innovative services in both 2017 and 2018. Likes for innovative services posts increased from 2016 through 2018 (*P*=.045 for the trend). By the end of 2018, we recorded 23,436 cumulative likes for posts, the highest number among medical centers in Taiwan.

**Conclusions:**

We achieved the largest number of Facebook fans among all medical centers in Taiwan. We would like to share our experience with other hospitals that might be interested in engaging in social media for future communications and interactions with their patients.

## Introduction

### Background

Branding plays an important role because a positive brand helps customers visualize and understand the products. A favorable brand image leads to positive outcomes in customer satisfaction, service quality, loyalty, and repurchasing intention [1-3]. Branding of health care is imperative in business marketing, especially in this digital era when most people search information daily on the internet. The International Telecommunication Union in its report on information and communication technologies for 2017 revealed that mobile broadband subscriptions grew by more than 20% during 2012 to 2017, with an expected global growth up to 4.3 billion users by the end of 2017 [4]. Social media apps are becoming increasingly popular because of the ease of sharing and disseminating information without barriers, both in time and in space [5]. Through social media, people can easily obtain information on health care services [6].

Consequently, the hospital corporate brand is increasingly focused on communications in social media. Digital marketing strategies need to be considered first. In an online world, corporate branding, and the brand experience, lives on the internet [7]. A Pew Research Center survey showed that 60% of US adults use the internet to search health care information, and 10% of them use social media to follow health care experiences shared by friends [8]. Another study reported that 34% of health scholars use social media, including online forums and message boards, to obtain information on health and wellness [9]. Therefore, the use of social media in health care will likely grow exponentially [10].

Increasingly more patients use social network sites to share their experiences with health care personnel and institutions [11]. People share their experiences and social support in particular with their families and friends via social media [12-16]. The main advantages of using social media in health care services, as perceived by patients, are the potential to improve doctor-patient communication, increase their understanding of health-related issues, and the ability to share experiences with other patients having similar health conditions [17]. Also, the ability to respond to patients’ needs and display timely messages on social media platforms means that hospitals could harness existing networks [18].

According to a survey on broadband internet usage in Taiwan, 89.4% of the 3155 respondents (extrapolated to Taiwan’s total of 18.81 million residents) had used the internet. Among 89.8% of social network or instant message service users, 75.6% used both of these services. Internet World Stats reported that Facebook has reached a penetration of 75.8% in Taiwan, the highest proportion of users in the world [19]. Another survey found that Facebook was also the most popular social medium (90.9%) among internet users, followed by LINE (a freeware app for instant communications on electronic devices; 87.1%), YouTube (60.4%), PTT (a terminal-based bulletin board system; 37.8%), and Instagram (32.7%) [20]. Therefore, we speculated that by deploying official Facebook fan pages, hospitals should be able to improve their exposure in the community, promote their reputations, and foster a better impression of these institutions. All of these can help ensure patient loyalty and recruit more patients.

Health care in Taiwan is managed centrally by the Bureau of National Health Insurance. By the end of 2017, the total number of insured people was 23.88 million, and the national health insurance coverage rate hovered around 99.7%. Also, 21,326 (92.8%) of the medical institutions in Taiwan had signed contracts with the National Health Insurance Administration of the Ministry of Health and Welfare. As of 2017, accreditation had been granted to 423 hospitals and 131 teaching hospitals. Based on the levels of accreditation, medical institutions are classified into 3 major categories: medical centers, regional hospitals, and local hospitals [21].

Prior to 2016, most of the hospital branding in Taiwan was focused on press conferences and press releases. Most hospitals assigned a single department to handle press conferences. Only 51.1% (n=213) of the hospitals had official Facebook fan pages as of 2017. Among them, academic medical centers tended to receive more likes than regional and local community hospitals [22]. The public sector receives relatively fewer resources from the government despite bearing a heavier burden than the private sector. Personnel and purchasing systems in public institutions are also less flexible, making them less competitive than private hospitals in providing medical services. Thus, how to restore or even to promote public recognition becomes a major issue for public hospitals.

### Objective

The aim of this study was to support the strategic imperative for promoting hospital branding by establishing a special branding team, led by top administrators of our hospital. Through constant monitoring of internet posts, we aimed to determine what audiences like and to continuously adjust the popular and useful types of posts on the hospital Facebook page and hospital website. The implementation of such cycles of monitoring and improvement, while successfully conducted in private businesses, has rarely been conducted in hospitals, and in particular in public hospitals, both in Taiwan and worldwide.

Here, we share our experience with other hospitals that might be interested in engaging in social media and using them for communications and interactions with their patients in the future.

## Methods

### Study Design

We conducted this study to support our strategic imperative for promoting hospital branding by establishing a special branding team, led by top administrators of the hospital. To ensure the effectiveness of this branding team, we obtained reference data from Google Analytics (Google LLC, Mountain View, CA, USA) and Insights reports from Facebook (Facebook, Inc, Menlo Park, CA, USA) for the year 2016 as the baseline for comparisons with data for 2017 and 2018.

### Strengthening Functions of Social Media

#### Study Setting

Taichung Veterans General Hospital is one of 19 medical centers in Taiwan. It was established in 1982, and in central Taiwan it is the only government medical center providing medical services to the public. It has 1569 beds, with high daily volumes of inpatient and outpatient services. Reflecting its performance quality, it is the only medical center awarded by the Healthcare Quality Improvement Campaign run by the Joint Commission of Taiwan in 2018 and received the Government Service Award from The National Development Council in 2019.

In the past, our institute, like most hospitals in Taiwan, issued regular news releases as the only tool to disseminate health information to the public. About 60 to 70 press conferences were held every year. To cope with recent marketing trends using social media, a branding team was established to strengthen online media marketing that included both Facebook and an internet website (Multimedia Appendix 1). Our aim here is to share our experiences with other institutes worldwide.

#### Establishing the Branding Team

**Figure 1:**
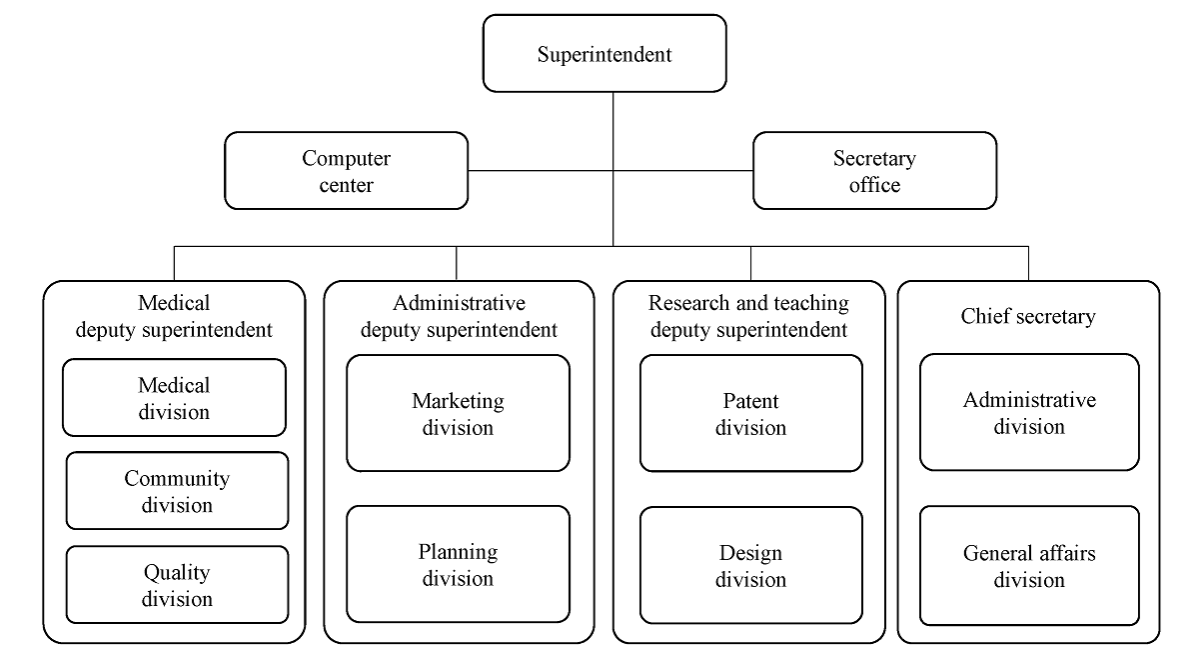
Taichung Veterans General Hospital branding team organization.

The branding team was first set up in 2016. Its main mission was to establish branding strategies and integrate segmental branding messages. Under the leadership of the hospital’s superintendent, 11 divisions were recruited to form this special task force (Figure 1). All divisions were required to draw up their own action plans and performance indicators. Top administrative officers oversaw and monitored task progress periodically.

#### Data Collection and Analyses

We obtained reference data for 2016 for comparison with data for 2017 and 2018. We analyzed statistical differences between the means using IBM SPSS version 21 (IBM Corporation). We applied simple linear regression models (2-sided) to test for the trend in the numbers of likes on the Facebook fan page, posts, and hospital website as obtained during the 3 years from 2016 to 2018.

## Results

### Outcome Performance Indicators

Each division of the branding team provided action plans together with the corresponding indicators, all of which we periodically monitored. We monitored a total of 52 action plans and indicators in each quarter and discussed the results at team meetings (Multimedia Appendix 2). We selected 4 main outcome performance indicators to reflect the effectiveness and outcome of the branding efforts (Table 1). Compared with 2016, the growth rate in Facebook fans was 61.2% (n=17,474) in 2017 and 116.2% (n=23,436) in 2018 (Multimedia Appendix 3). Similarly, website visits increased by 4.8% (n=9,389,164) in 2017 and 33.1% (n=11,930,020) in 2018 (Multimedia Appendix 4).

The branding team also formed a LINE (LINE Corporation, Tokyo, Japan) group as a communication channel for patient referrals. There were 489 (in 2017) and 719 (in 2018) primary physicians participating in the LINE group with a growth rate of 100.4% in 2017 and 195.1% in 2018 (Multimedia Appendix 5). To obtain real-time information from the hospital, we invited all primary physicians to join our Facebook fan page. In addition, the branding team asked each medical group to set up peer support groups and to organize at least two activities each year. Reports of the activities held by 46 peer support groups in 2018 (Multimedia Appendix 6) were posted on Facebook. Patients and their families were also invited to join the hospital’s fan page.

**Table 1:** Outcome performance indicators monitored by the branding team.

Indicators	Year				Growth rate (%)^a^		*P* value
	2016	2017	2018	2017		2018	
Number of likes on Facebook fan page	10,841	17,474	23,436	61.2		116.2	.02
Number of visits to the official website	8,963,097	9,389,164	11,930,020	4.8		33.1	.25
Number of clinic physicians joining LINE^b^	244	489	719	100.4		195.1	.01
Number of peer support groups	31	45	46	45		48	.30

^a^Growth rate is defined as the value of 2017 or 2018 minus that of 2016, and the result expressed as a percentage of 2016.

^b^A freeware app for instant communications on electronic devices.

### General Public and Patient Engagement

According to the Facebook Insights report for 2018, 54.54% (227,989/417,988) of target audiences and 60.42% (14,160/23,436) of Facebook fans were women. Most were between 25 and 44 years of age (199,906/417,988, 47.83%). Based on Google Analytics, more women (778,992/1,311,605, 59.39%) than men visited the hospital website. Website viewers were mostly between 25 and 44 years of age (778,668/1,289,988, 60.36%) (Table 2).

**Table 2:** Sex and age group distributions on the Facebook fan page and hospital website in 2018, according to Facebook Insights reports Google Analytics.

Characteristics		Facebook fan page, n (%)		Website visits, n (%)
		Target audiences	Fans	
**Sex**				
	Female	227,989 (54.54)	14,160 (60.50)	778,992 (59.39)
	Male	189,999 (45.46)	9276 (39.50)	532,613 (40.61)
	Total	417,988 (100.00)	23,436 (100.0)	1,311,605 (100.00)
**Age group (years)**				
	13-17	1790 (0.43)	166 (0.71)	—^a^
	18-24	41,699 (9.98)	2062 (8.80)	170,292 (13.20)
	25-34	100,582 (24.06)	5479 (23.38)	424,779 (32.93)
	35-44	99,324 (23.76)	6441 (27.48)	353,889 (27.43)
	45-54	70,072 (16.76)	4675 (19.95)	202,188 (15.67)
	55-64	71,287 (17.05)	3218 (13.73)	131,884 (10.22)
	≥65	33,234 (7.95)	1395 (5.95)	6956 (0.54)
	Total	417,988 (100.00)	23,436 (100.0)	1,289,988 (100.0)

^a^Not available.

Table 3 shows that the number of posts increased from 461 in 2016 to 595 in 2017 (growth rate of 29.1%) and 566 in 2018 (growth rate 22.8%). The number of videos posted was 16 in 2016, increasing to 49 in 2018 and to 90 in 2018 (*P*=.04 for the trend). The number of likes almost doubled in 2017 (n=99,262) over 2016 and stayed at approximately the same level in 2018 (n=91,337; *P*=.42, for the trend). Increments were similarly marked for comment messages, which increased nearly threefold in 2017 (n=2931) and fourfold in 2018 (n=4559; *P*=.02 for the trend). Post sharing also doubled in both years (n=1755 in 2016, n=4783 in 2017, n=4629 in 2018; *P*=.36 for the trend).

**Table 3:** Facebook Insights reports for posts made from 2016 to 2018.

Items	Year, n				Growth rate, %^a^			*P* value
	2016	2017	2018	2017		2018		
Posts	461	595	566	29.1		22.8	.47	
Videos	16	49	90	206		463	.04	
Post likes	46,014	99,262	91,337	115.7		98.5	.42	
Comment messages	1127	2931	4559	160.1		305.2	.02	
Post sharing	1755	4783	4629	91.9		85.6	.36	

^a^Growth rate is defined as the value of 2017 or 2018 minus that of 2016, and the result expressed as a percentage of 2016.

### Post Categories and the Analysis of Likes on Facebook

In total, 1622 posts were provided by each division of the branding team from 2016 to 2018. We divided posts according to their content characteristics into 14 unique post themes and hashtags in the text (Multimedia Appendix 7).

To identify what types of posts audiences liked, we classified the 14 themes into 5 groups for analysis: innovative service, media reports, activity information, patient gratitude letter, and health education information. Table 4 shows that the type of post with the highest number of likes was media reports in 2016, being replaced by innovative service in both 2017 and 2018. The mean number of likes for innovative service posts rose significantly from 2016 through 2018 (*P*=.045 for the trend). The numbers of likes for health education information posts remained the lowest for all 3 years.

**Table 4:** Likes per post category on the hospital Facebook fan page from 2016 to 2018.

Type of post	2016					2017					2018					*P* value
	Likes, n	Posts, n	Mean^a^	Rank	Likes, n		Posts, n	Mean^a^	Rank	Likes, n		Posts, n	Mean^a^	Rank		
Innovative service	4299	39	110	2	9377		40	234	1	19,539		59	331	1	.045	
Media reports	7074	57	124	1	23,796		163	146	4	33,374		151	221	2	.2	
Activity information	20,972	198	106	3	42,599		212	201	2	16,882		136	124	4	.89	
Patient gratitude letter	3862	42	92	4	7214		46	157	3	6307		46	137	3	.53	
Health education information	9807	125	78	5	16,276		134	121	5	15,235		174	88	5	.86	

^a^Average number of likes per post (number of likes divided by number of posts).

**Figure 2:**
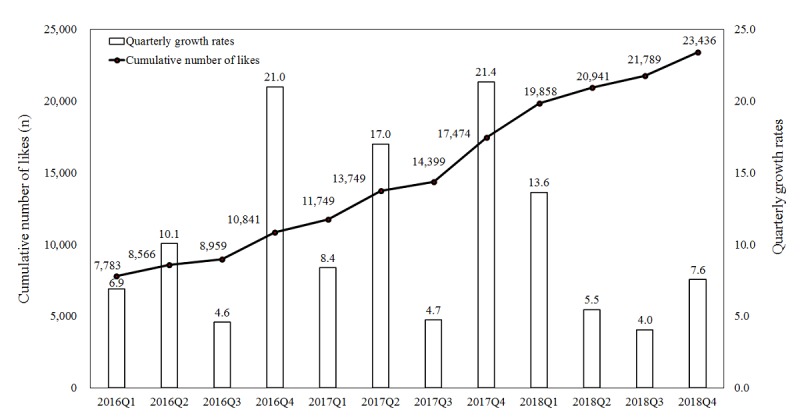
Cumulative numbers and quarterly growth rates of likes on the Facebook fan page from 2016 to 2018. Q: quarter.

Figure 2 shows the cumulative number of likes since 2016. The growth rate from the first quarter to the second quarter was 10.1% in 2016, 17.0% in 2017, and 5.5% in 2018. The growth rate from the third quarter to the fourth quarter was 21.0% in 2016, 21.4% in 2017, and 7.6% in 2018. Some of the increases in the numbers of likes were apparently related to promotional activities during festivals, as well as interactions with fans. For example, fan interaction activities were intensified during the Chinese New Year and Christmas season.

## Discussion

### Principal Findings

Our main finding is that a public medical center in Taiwan such as ours can, through the efforts of a branding team led by top administrators, gain Facebook fans. We attained the highest number of Facebook fans among medical centers in Taiwan, with a 116.2% increase over 2 years. We believe that the factors contributing to this success were (1) the interconnected Facebook and hospital webpages, (2) active participation of the branding team with timely provision of attractive posts and videos, (3) strengthened interactions with online visitors, (4) fast responses to users’ queries and messages, and (5) increased online streaming.

### Functions of the Branding Team

With strong support from the top administrators, the branding team was able to play a key role in implementing strategic marketing functions. We identified several distinguished medical services as strategic features through interdepartmental communications. Results suggested that, in this digital era, targeting social media is an effective approach for promoting health consumer education, health care group communication, and brand awareness. Our Facebook audience not only came from the general public, but also from strategically invited peer support groups and primary physicians. Given the importance of brand management and extraction of associated values, health service organizations should diligently attend to branding initiatives. Enhanced values can be derived by addressing nontraditional brand elements that provide unique opportunities to facilitate institutional viability and vitality [23].

Hospitals use the Facebook platform as an inexpensive way to educate people on topics of health and well-being, and to communicate different types of information and news to the general public. Based on more than 1700 Facebook posts from 17 hospitals in the United States, Kordzadeh and Young [24] identified 13 unique health social media post themes and classified them into 3 thematic groups that included announcing, sharing, and recognizing activities. The most frequently used theme was the sharing of health information, which appeared in 35.8% (424/1184) of the posts. Such posts provided health tips and advice to community members [24]. In our study, we classified 14 themes into 5 groups: innovative service, activity information, media reports, health education information, and patient gratitude letter. In 2016, the most popular post theme was media reports. The main reason is that this information was generally linked to television news reports rather than plain-text posts. Since 2017, for proposing innovative service, health tips and advice were strategically posted in various forms, such as online streaming or videos. Due to the good strategy, innovative service became the most popular post theme. The number of likes for health education information remained the lowest for all 3 years. The main reason may be related to the plain-text form by which the theme was posted. At present, we are systematically replacing plain texts with more interesting online streams or videos.

In addition, to expand our brand and intensify fan interaction, we sent branded gifts such as management books we had published, as well as mugs, ties, purses, and pens carrying our hospital logo, during online streaming. Since many people nowadays prefer watching videos to reading texts, the branding team decided to improve video streaming in real time and to strengthen links to other online media. Probably as the consequence of the above measures, the number of likes rose to make ours the most popular Facebook fan page among all medical centers in Taiwan.

### Special Features of Our Facebook Page

Using social media not only promotes marketing, but also upgrades care for patients and their families, enhances health consumer education, advances medical research, and expands brand awareness [25]. Facebook pages also serve as a tool for patient empowerment and allow for intercommunication between physicians and patients. Given the high volume of posts, it is imperative that the information provided be accurate and in accordance with the medical advice of physicians [26]. Other studies used Facebook for disease surveillance [27] and health interventions [28]. Facebook likes can reflect users’ preference, and thus can help predict health-related behaviors [29,30]. A previous study in 2014 reported that 99.4% (3351/3371) of hospitals in the United States had established Facebook pages. The use of social media varied according to the different characteristics of hospitals such as their size, urban location, and whether they were private nonprofit or teaching hospitals. All these factors affected their levels of activity on Facebook [18]. Another study of 12 Western European countries in 2012 found that 67.0% (585/873) of their hospitals had Facebook fan pages [31].

The penetration rate of Facebook in Taiwan is 82%, which is the highest in the world [32]. Despite this, only 51.1% of the hospitals have official Facebook fan pages. Furthermore, in comparison with other commercial Facebook fan pages, the numbers of likes are relatively low on hospital Facebook fan pages in Taiwan [22]. Social media is cheaper than conventional marketing, but its effects are enormous. For public hospitals with a restricted marketing budget, promotion through social media is a good investment approach. To strengthen hospital branding and to synchronize with the latest news, our results showed that Facebook and the hospital website are better interconnected. In addition, for better results, information should be posted quickly, within hours of events, and query comment messages should be answered as soon as possible.

### Limitations

Our study provided insights into the ways our hospital had established a special team to promote branding by recruiting fans to our hospital Facebook page. However, our study had the following limitations. First, we reported and analyzed the methods of only our own hospital, and did not collect data from other hospitals for comparison. Second, social media and channels are changing rapidly. The use rate of different social media in 2019 could have been different from 2017. We have already expedited the construction of a LINE group in our hospital because of its increasing popularity over Facebook. Third, our Facebook fan page was established in 2012, while we obtained the reference data only in 2016 when we first started our Facebook drive. Fourth, since in Taiwan Facebook and mobile phones had been gaining in popularity from 2016 onward, we implemented these strategies for only 3 years. Details on longitudinal trends remain to be studied.

### Conclusions

Our branding team, led by the hospital’s top officers, successfully implemented several strategies that achieved the most popular Facebook fan page among Taiwan hospitals. Strategies we used were powerful in providing information on time and in promoting better medical services. Our unique experience in Facebook management may lay the groundwork for hospitals’ use of social media platforms to improve patient interactions and health care outcomes.

## Appendix

Multimedia Appendix 1 Facebook fan page and hospital website of Taichung Veterans General Hospital.

Multimedia Appendix 2 The 52 performance indicators monitored, by branding team.

Multimedia Appendix 3 Counts of visitors to the Facebook fan page per month from 2016 to 2018.

Multimedia Appendix 4 Counts of visitors to the hospital website per month from 2016 to 2018.

Multimedia Appendix 5 LINE groups for primary physicians.

Multimedia Appendix 6 Peer support group webpage.

Multimedia Appendix 7 Themes of Facebook posts from 2016 to 2018.
