# Supplementary figures and images for: A Strategic Imperative for Promoting Hospital Branding: Analysis of Outcome Indicators
Source: Interact J Med Res. 2020 Jan 22;9(1):e14546. doi: 10.2196/14546 (PMC7003120; doi:10.2196/14546)

Facebook website URL: <https://www.facebook.com/vghtc/>

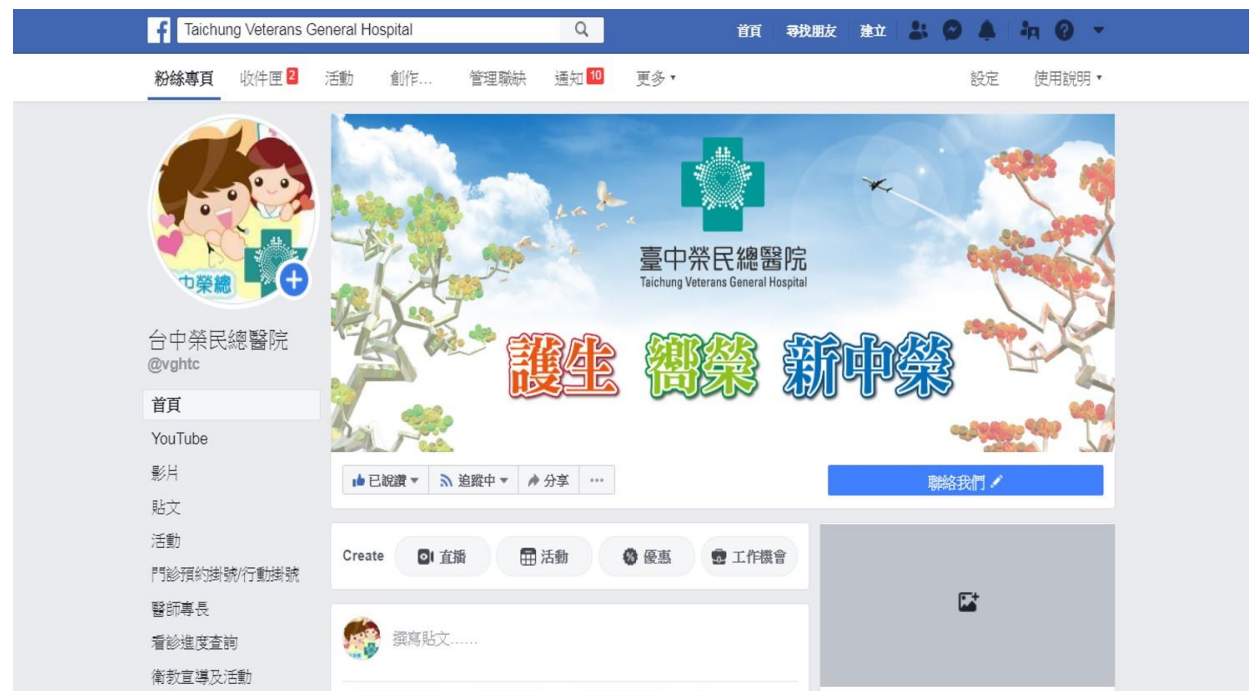

Hospital website URL: <http://www.vghtc.gov.tw/>

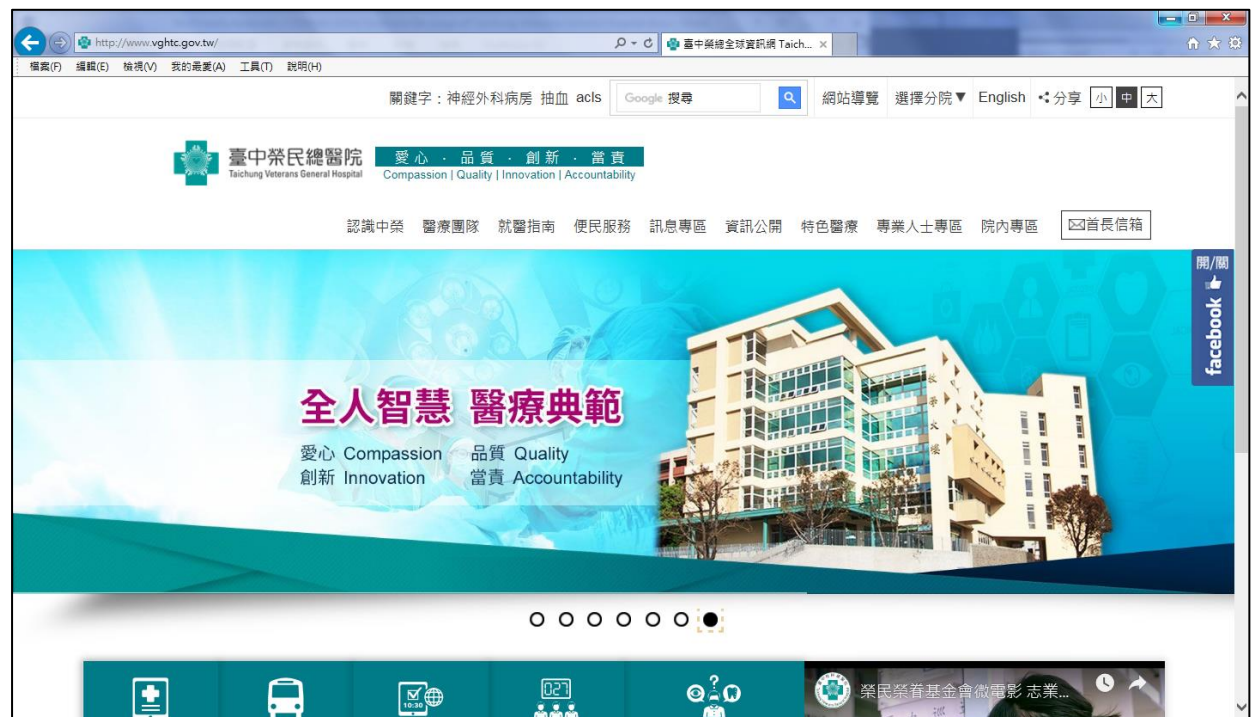

Supplement: Multimedia Appendix 1 [file ijmr_v9i1e14546_app1.pdf]
