# Supplementary material for: A Strategic Imperative for Promoting Hospital Branding: Analysis of Outcome Indicators
Source: Interact J Med Res. 2020 Jan 22;9(1):e14546. doi: 10.2196/14546 (PMC7003120; doi:10.2196/14546)
Supplement: Multimedia Appendix 2 [file ijmr_v9i1e14546_app2.pdf]

Division	Indicators
Secretary office	1. Facebook “Likes” quarterly growth rate
	2. Facebook video number of monthly uploads
	3. Making branding story cards
	4. Collecting touching stories
	5. Media interviews
	6. Clinic physician joining number of LINE
Computer center	7. Simplified website layout
	8. Visual presentation of the website
	9. Insights analysis of the website activities
	10. Compliance of website with web design

Division	Indicators
Medical	11. Self-paid income growth rate
	12. Integrated marketing documents and photos
	13. Workflow standardization
Medical (BOHS)	14. BOHS referral health management center
	15. BOHS referral outpatient/inpatient/emergency
	16. Medical service volume (BOHS)
	17. Number of firms visited
	18. BOHS satisfaction survey
	19. Percentage of completed firm health plan (BOHS)
	20. Reduce the number of staff of firms without health plans

BOHS: Basic occupational health services

Division	Indicators
Community (Labor Physical Checkup)	21. Securing 2016 labor quality inspection certification
Community (Nurse)	22. Consultation volume of 24-hours health call center
	23. Action guardian APP knowledge-based architecture (oral cancer patients)
	24. Action guardian APP system architecture (oral cancer patients)
	25. Fourteen days hospitalization rates of discharged patients
	26. Three days of emergency rates of discharged patients
Quality	27. Number of peer support groups
Planning	28. Organizing marketing-related lectures
	29. Production of new version hospital profile video
	30. Complete the corporate identity system plan
	31. Satisfaction analysis

Division	Indicators
Marketing	32. Press conferences for medical topics
	33. Press conferences for plane or electronic reports
	34. Press conferences for mainstream media
	35. Promotion of health education for peer support groups
	36. Promotion of health education for health lecture
	37. Social responsibility for vulnerable groups performance
	38. Social responsibility for patients care activity
	39. Social responsibility for micro film
	40. Media reports

Division	Indicators
Patent	41. Talent cultivation (organize patent course)
	42. Industrial-academic cooperation promotion
	43. R&D (established a 3D printing center)
	44. R&D (laundry factory automation)
	45. R&D (participate in National Innovation Award competition)
Design	46. Subscription rates of TCVGH electronic journal
	47. Articles online views of TCVGH electronic journal
Administrative	48. Annual meetings of retired friendly associates
	49. Annual cadre meeting for retired friendly associates
	50. Annual dinner events for retired friendly associates
	51. Monthly updates of the retiree's service website
General affairs	52. Organize a photo contest

R&D: Research & Development
TCVGH: Taichung Veterans General Hospital
