# Supplementary material for: A Strategic Imperative for Promoting Hospital Branding: Analysis of Outcome Indicators
Source: Interact J Med Res. 2020 Jan 22;9(1):e14546. doi: 10.2196/14546 (PMC7003120; doi:10.2196/14546)

Medical referral communication group has 493 physicians in this group

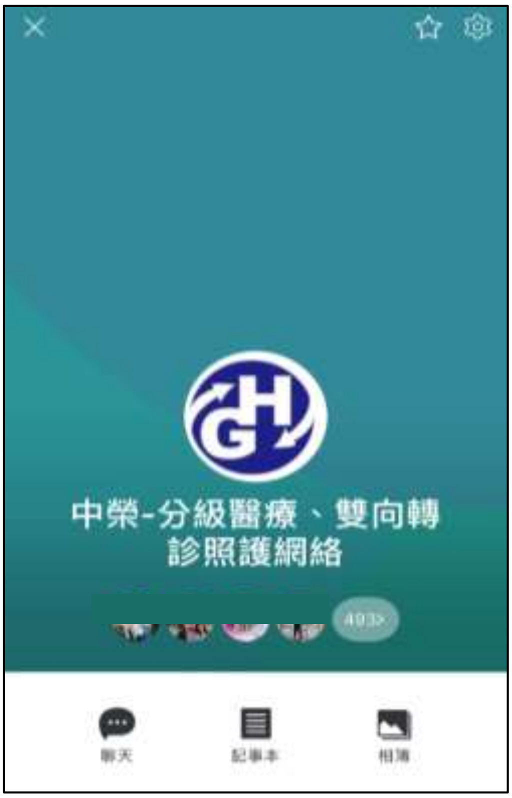

Pediatric referral communication group has 226 physicians in this group

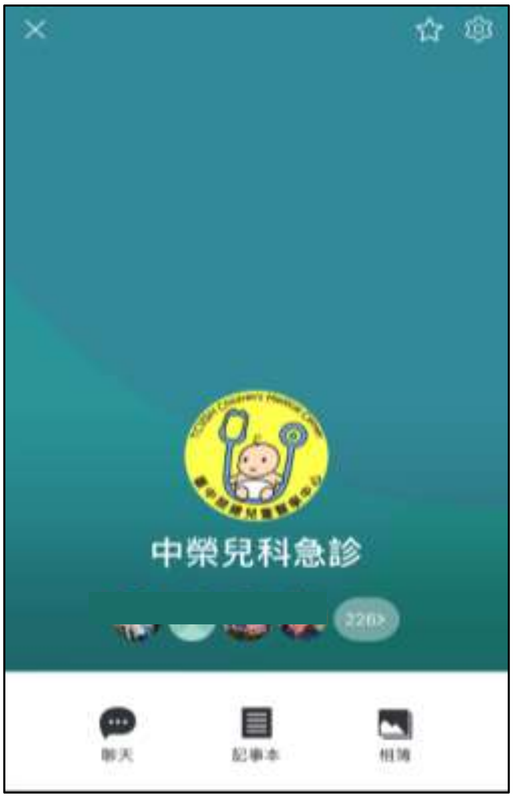

Supplement: Multimedia Appendix 5 [file ijmr_v9i1e14546_app5.pdf]
