# Supplementary material for: A Strategic Imperative for Promoting Hospital Branding: Analysis of Outcome Indicators
Source: Interact J Med Res. 2020 Jan 22;9(1):e14546. doi: 10.2196/14546 (PMC7003120; doi:10.2196/14546)
Supplement: Multimedia Appendix 6 [file ijmr_v9i1e14546_app6.pdf]

URL:

https://www.vghtc.gov.tw/UnitPage/UnitContentView?WebMenuID=9a7647c8-4a33-41d1-b771-788e8bdb57c1&UnitID=ecced4da-47b6-4df4-8dd8-76a1b9b3b5a0&UnitDefaultTemplate=1

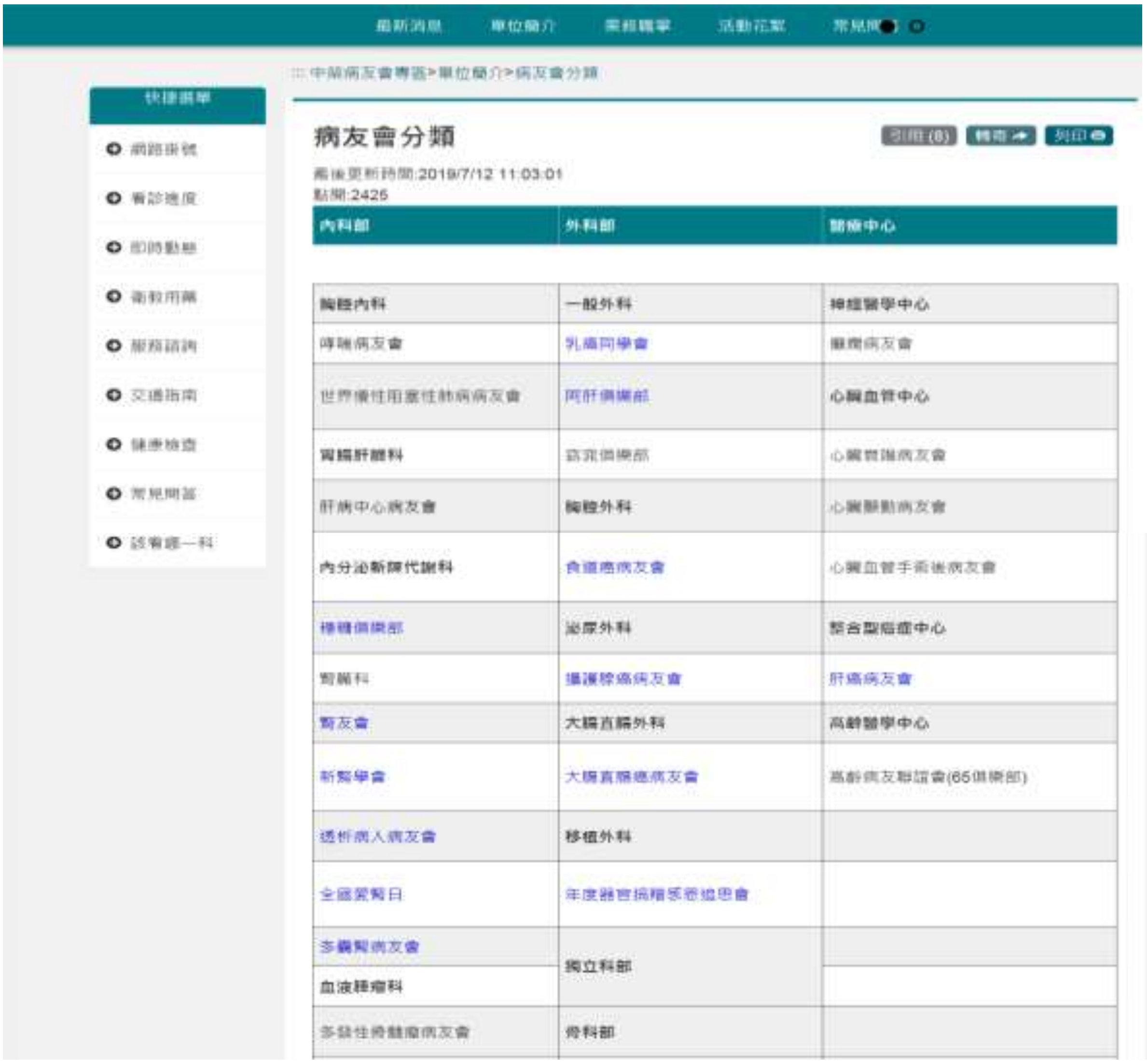

骨髓纖維化病友會	骨質疏鬆病友會	
過敏免疫風濕科	**婦女醫學部**	
台灣免疫風濕疾病關懷協會	間質性膀胱炎病友聯誼會	
**感染科**	卵巢癌病友會	
愛滋病友會	**兒童醫學部**	
**呼吸治療科**	兒童糖尿病病友會	
哮喘病友會	兒童血友病病友會	
世界慢性阻塞性肺病病友會	兒童癲癇病友會	
	兒童心臟病童病友會	
	癌症病童病友會	
	威廉斯氏症病友會	
	法布瑞氏症病友會	
	早產兒回娘家	
	**精神部**	
	精神病患病友會	
	**耳鼻喉頭頸部**	
	無喉語言復健病友會	
	**家庭醫學部**	
	安寧遺族關懷	
	**口腔醫學部**	
	口腔癌症病友會	
	**皮膚科**	
	乾癬病友會	
	**復健科**	
	康復之路病友會	
